# Assessing teamwork perceptions and challenges in a South African district hospital

**DOI:** 10.4102/safp.v68i1.6290

**Published:** 2026-03-31

**Authors:** Willem M. van Tonder, Matthew A. Benedict, Francois C. van Rooyen

**Affiliations:** 1 Department of Family Medicine, School of Clinical Medicine, Faculty of Health Sciences, University of the Free State, Bloemfontein, South Africa; 2 Department of Biostatistics, School of Biomedical Sciences, Faculty of Health Sciences, University of the Free State, Bloemfontein, South Africa

**Keywords:** teamwork, healthcare, professionals, perception, district hospital, TeamSTEPPS, situational monitoring, staff

## Abstract

**Background:**

The South African healthcare sector faces many challenges that jeopardise safety and quality of care. Amid these challenges, effective teamwork is essential to promote safe and quality healthcare services. This study aimed to measure the overall teamwork perception among healthcare professionals at National District Hospital (NDH) and compare differences in perceptions across healthcare disciplines and departments.

**Methods:**

An analytical cross-sectional study was conducted using a questionnaire distributed among all healthcare professionals at NDH. The questionnaire consisted of a demographic section and the validated Team Strategies and Tools to Enhance Performance and Patient Safety (TeamSTEPPS) 2.0 Teamwork Perceptions Questionnaire (T-TPQ).

**Results:**

The overall mean teamwork perception score among healthcare professionals was 3.7 (74%). While no statistically significant difference was found across disciplines (*p* = 0.434) and departments (*p* = 0.066), hospital-wide analysis identified situational monitoring (72%) and leadership (73%) as the weakest domains. Conversely, communication (79%) was the strongest domain. Critical unit-specific deficits were identified in the leadership domain of two departments.

**Conclusion:**

The overall perception of teamwork was acceptable. However, targeted interventions are required to improve situational monitoring hospital-wide and to address the leadership challenges found in some departments. These findings provide evidence-based direction for quality improvement within this specific district hospital setting. The T-TPQ is a valuable tool that all institutions should utilise to assess their healthcare services independently and identify strategies to improve teamwork.

**Contribution:**

This study enhances the limited existing literature on teamwork and its assessment within healthcare settings, particularly within the context of South African district health services.

## Introduction

South African healthcare professionals are required to provide comprehensive services for patients. The primary healthcare sector also contends with a complex fourfold burden of disease. This is compounded by systematic challenges within the district health system, including staff shortages, questionable infrastructure, and regular stockouts of essential supplies.^1^ Given the complexity, providing safe and high-quality patient care is a feat that cannot be dealt with by any single individual.^2^ Therefore, teamwork, the collaborative effort of multiple healthcare professionals across disciplines focussing on a common goal, is essential to mitigate these risks and achieve a satisfactory patient outcome.^3,4^

The beneficial effect of teamwork is well documented. It enhances client outcomes and satisfaction, decreases burnout among staff, improves staff retention, optimises resource use, and creates a more enjoyable work environment.^2,3,4,5,6,7^ Conversely, suboptimal teamwork has been identified as an independent contributor to health system failures and patient harm.^8^ Thus, many countries rate teamwork as a core competency and have begun improvement efforts by measuring the current state of teamwork perception among healthcare professionals.^7,8,9,10,11^ Despite the global focus, limited published literature exists on teamwork assessment within the context of South African district hospitals, representing a substantial knowledge gap.

Addressing this research gap, this study aimed to measure the overall teamwork perception among healthcare professionals at National District Hospital (NDH) by utilising the validated Team Strategies and Tools to Enhance Performance and Patient Safety (TeamSTEPPS) 2.0 Teamwork Perceptions Questionnaire (T-TPQ).^12^ The T-TPQ assesses five core components of teamwork: team structure, leadership, situational monitoring, mutual support, and communication. Within this framework, leadership is evaluated not as a separate administrative tier, but as an integrated function that coordinates and supports the team from within. By identifying both challenges and areas of excellence, this research serves to prioritise targeted interventions to promote team effectiveness and patient safety within the district hospital setting.^8,10,11^

## Research methods and design

### Study design

This study employed an analytical cross-sectional design.

### Study setting

National District Hospital is a 197-bed facility in Bloemfontein, Free State Province, South Africa. The facility provides district-level services to the greater Bloemfontein region, serving a community of about 567 000.^13^

### Study population and sampling strategy

The study population consisted of 256 healthcare professionals delivering direct professional patient care at NDH. No sampling was done, as the entire study population was targeted for participation.

The inclusion criteria:

- Current registration with the applicable regulatory bodies (South African Council for Social Service Professions, South African Nursing Council, Health Professions Council of South Africa, or the South African Pharmacy Council).
- Delivering direct primary healthcare to patients of NDH.

The exclusion criteria:

- Students in training.
- Doctors only doing after-hour calls.
- Unit managers or departmental heads.
- Supportive and administrative personnel.
- Healthcare professionals absent from duty during the data collection period.
- Units with only one staff member (to ensure teamwork could be assessed).

### Data collection

The Team Strategies and Tools to Enhance Performance and Patient Safety (TeamSTEPPS) 2.0 Teamwork Perceptions questionnaire (T-TPQ) was used. This is a validated tool that categorises core components of effective teamwork into five domains: team structure, leadership, mutual support, communication, and situational monitoring (see Table 1 for domain definitions).^8,14,15,16,17,18,19^ The T-TPQ assesses these domains with seven statements per domain on a 5-point Likert scale, where 1 = ‘Strongly disagree’ and 5 = ‘Strongly agree’.^10,12^

**TABLE 1:** Overview of TeamSTEPPS 2.0 Teamwork Perceptions Questionnaire teamwork domains and focus areas.

Domain	Key focus of statements or questions
Team structure	Understanding team size, membership, roles, responsibilities, and the common goal.
Leadership	The ability of leaders to coordinate activities, share information, resolve conflicts, and manage resources.
Situational monitoring	Actively scanning the environment, cross-monitoring team members, and maintaining shared awareness of the situation.
Mutual support	Aiding teammates, offering constructive feedback, and advocating for patient safety.
Communication	The effective exchange of information among team members using standardised tools and clear language.

*Source*: Adapted from the TeamSTEPPS 2.0 Teamwork Perceptions Questionnaire (T-TPQ) domains. Kakemam E, Rouzbahani M, Rajabi MR, Roh YS. Psychometric testing of the Iranian version of the TeamSTEPPS teamwork perception questionnaire: A cross-cultural validation study. BMC Health Serv Res. 2021;21:705. https://doi.org/10.1186/s12913-021-06739-z,^10^ Agency for Healthcare Research and Quality. Teamwork Perceptions Questionnaire (T-TPQ) & Manual: Team Strategies & Tools to Enhance Performance & Patient Safety (TeamSTEPPS) 2.0 [homepage on the Internet]. Rockville, MD: AHRQ; 2014 [cited 2024 Sep 12]. Available from: https://www.ahrq.gov/teamstepps/instructor/tools.html^12^

The questionnaire, available only in English as per the hospital policy on communication, consisted of statements confirming participants’ eligibility to participate, two relevant demographic questions, and the T-TPQ. Questionnaires were distributed to all healthcare professionals over a 5-day period (08 April 2024 – 12 April 2024).

To maximise response and avoid disrupting service delivery, time slots covering both day and night shifts were arranged with each department in advance. Departmental heads or supervisors indicated the number of staff available to participate. Questionnaires were completed in departmental tearooms to ensure adequate privacy, anonymity, and voluntary participation. Complete questionnaires were folded and placed into a sealed box, thereby implying informed consent.

### Pilot study

A pilot study was conducted 3 months before the main study with 10 participants (including nursing staff, allied health professionals, and doctors) at NDH to test the questionnaire. The biostatistician from the Department of Biostatistics, University of the Free State (UFS), confirmed that these responses could be included in the results as no adjustments were made to the questionnaire content.

### Data analysis

The principal researcher transferred the data from the paper-based questionnaires to the *Protection of Personal Information Act (POPIA)*-compliant REDCap platform hosted on the UFS’s secure server. Incomplete questionnaires were discussed individually with the allocated biostatistician to determine usability. The biostatistician accessed and analysed the data using SAS statistical software (version 9.4). The numerical variables were summarised by means, standard deviations (s.d.), minimum, and maximum, and the categorical variables were summarised by frequencies and percentages. Differences between numerical variables were evaluated using the Wilcoxon Two-Sample test for unpaired data. A *p*-value of *p* < 0.05 was taken to be statistically significant.

### Ethical considerations

Full ethical approval was obtained from the Health Sciences Research Ethics Committee (HSREC) of the University of the Free State. The ethical approval number is UFS-HSD2023/1664/2811. Permission was also obtained from the Head of the Free State Department of Health. The Agency for Healthcare Research and Quality (AHRQ), granted permission to use the TeamSTEPPSO 2.0 Teamwork Perceptions Questionnaire (T-TPQ) and the TeamSTEPPS^®^ 2.0 Teamwork Perceptions Questionnaire (T-TPQ) Manual. Implied informed consent was obtained from all participants to promote anonymity and confidentiality.

## Results

**FIGURE 1:**
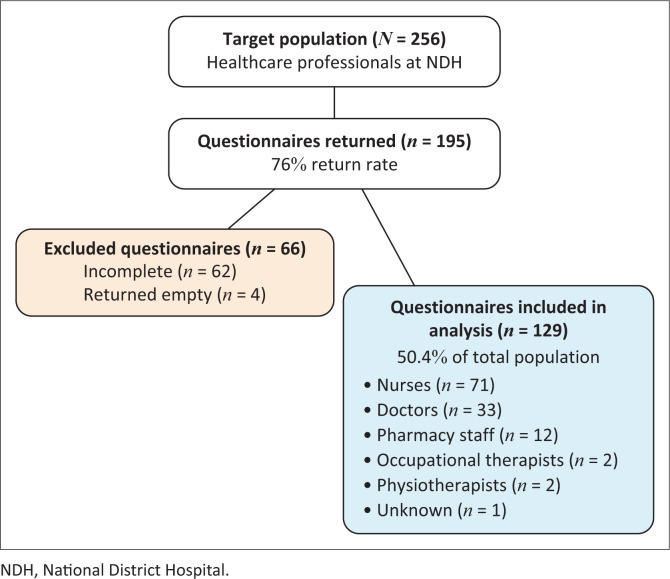
Flowchart of participants and questionnaire data.

A total of 195 questionnaires were returned, resulting in a 76% return rate for the target population (*N* = 256). Of these, 129 questionnaires were sufficiently complete for analysis, representing 50.4% of the total study population. The remaining 66 questionnaires were excluded, consisting of 62 too incomplete for analysis and 4 returned empty. (See Figure 1 for a detailed data flow breakdown.)

The final analysed sample (*n* = 129) comprised predominantly of nursing staff (*n* = 71) and doctors (*n* = 33). The social work (*n* = 2) and psychology (*n* = 1) departments were excluded from the study to protect participant anonymity, as their small size and the inclusion of departmental heads would have compromised confidentiality.

### Overall teamwork perception

The overall mean teamwork perception score among healthcare professionals were 3.7 (74%). Table 2 shows the overall teamwork perception results for the five teamwork domains.

**TABLE 2:** Overall teamwork perception among healthcare professionals at National District Hospital (*n* = 129).

Teamwork domains	Mean		s.d.	Median	Min.	Max.
	Score (out of 5)	%				
Communication	3.9	79	0.7	4.0	1.4	5.0
Team structure	3.7	75	0.8	3.9	1.4	5.0
Mutual support	3.7	74	0.8	3.7	1.7	5.0
Leadership	3.6	73	0.9	3.7	1.0	5.0
Situational monitoring	3.6	72	0.8	3.7	1.3	5.0
TOTAL	3.7	74	0.7	3.7	1.9	5.0

s.d., standard deviation; Min., minimum; Max., maximum.

All domains scored relatively similarly, between 3.6 (72%) and 3.9 (79%). The communication domain scored the highest (3.9; 79%). The situational monitoring domain scored the lowest (3.6; 72%), closely followed by the leadership domain (3.6; 73%).

### Differences in teamwork perception

The total analysed sample was *n* = 129; however, for inter-disciplinary analysis, one questionnaire was excluded due to the participant not indicating their healthcare discipline, resulting in an *n* = 128.

Table 3 shows the difference in perception of teamwork among healthcare disciplines. There was no significant difference in the perception of teamwork among the healthcare disciplines (*p* = 0.434). The highest mean score for the perception (4.1; 81%) was reported by the dieticians (*n* = 2) and occupational therapists (*n* = 2), and the lowest scores were recorded by doctors (3.5; 70%) and pharmacy staff (3.6; 71%).

**TABLE 3:** Differences in overall teamwork perception across healthcare disciplines (*n* = 128).

Healthcare disciplines	*n*	Mean		s.d.	Median	Min.	Max.
		Score (out of 5)	%				
Dieticians	2	4.1	81	0.6	4.1	3.6	4.5
Occupational therapist	2	4.1	81	0.4	4.1	3.8	4.3
Radiology staff	6	3.9	78	0.5	3.9	3.3	4.8
Physiotherapists	2	3.8	77	0.2	3.8	3.7	4.0
Nursing staff	71	3.8	76	0.6	3.8	2.4	5.0
Pharmacy staff	12	3.6	71	1.1	3.8	1.9	4.9
Doctors	33	3.5	70	0.7	3.4	2.2	4.8
TOTAL	128	3.7	74	0.7	3.8	1.9	5.0

s.d., standard deviation; Min., minimum; Max., maximum.

Table 4 shows the differences in the perception of teamwork among healthcare departments. There was no significant difference in the perception of teamwork among the different healthcare departments (*p* = 0.066). The Thusong trauma centre, NDH’s unit for survivors of abuse, scored the highest (4.7; 94%), while the lowest scores were recorded by the outpatient department (3.3; 66%) and casualties (3.5; 70%).

**TABLE 4:** Differences in overall teamwork perception across healthcare departments (*n* = 129).

Healthcare departments	*n*	Mean		s.d.	Median	Min.	Max.
		Score (out of 5)	%				
Thusong trauma centre	2	4.7	94	0.4	4.7	4.4	4.9
Theatre	5	4.4	87	0.4	4.4	4.0	4.8
Dietician department	2	4.1	81	0.6	4.1	3.6	4.5
Occupational health	2	4.1	81	0.4	4.1	3.8	4.3
Paediatric ward	8	4.0	80	0.3	3.9	3.7	4.4
Radiology department	6	3.9	78	0.5	3.9	3.2	4.8
Physiotherapy	2	3.8	77	0.2	3.8	3.7	4.0
Maternity ward	16	3.7	75	0.5	3.6	3.0	5.0
Adult wards	37	3.7	74	0.8	3.7	2.2	4.9
Pharmacy	12	3.6	71	1.1	3.8	1.9	4.9
Casualty	34	3.5	70	0.7	3.4	2.3	4.9
Outpatient department	3	3.3	66	0.4	3.1	3.0	3.8
TOTAL	129	3.7	74	0.7	3.7	1.9	5.0

s.d., standard deviation; Min., minimum; Max., maximum.

### Domain-specific findings

Table 5 and Table 6 summarise the mean scores, highlighting specific areas of strength and weakness across disciplines and departments.

**TABLE 5:** Mean teamwork domain scores across healthcare disciplines (*n* = 128).

Healthcare disciplines	*n*	Team structure		Leadership		Situational monitoring		Mutual support		Communication	
		Score(out of 5)	%	Score(out of 5)	%	Score(out of 5)	%	Score(out of 5)	%	Score(out of 5)	%
Dieticians	2	4.4	87	4.4	87	3.7	74	3.7	74	4.1	83
Radiology staff	6	4.1	83	4.0	80	3.6	72	4.0	79	3.9	77
Occupational therapists	2	4.1	81	4.5	90	3.8	76	4.1	81	3.9	77
Physiotherapist	2	3.9	77	2.2	44	4.0	80	4.5	90	4.6	93
Nursing staff	71	3.8	76	3.6	72	3.7	74	3.8	76	4.1	81
Pharmacy staff	12	3.5	71	3.6	72	3.3	67	3.4	68	3.9	78
Doctors	33	3.5	69	3.6	73	3.4	68	3.5	69	3.6	72
TOTAL	128	3.7	75	3.6	73	3.6	72	3.7	74	3.9	79

**TABLE 6:** Mean teamwork domain scores across healthcare departments (*n* = 129).

Healthcare departments	*n*	Team structure		Leadership		Situational monitoring		Mutual support		Communication	
		Score (out of 5)	%	Score(out of 5)	%	Score(out of 5)	%	Score(out of 5)	%	Score(out of 5)	%
Adult wards	37	3.7	74	3.7	74	3.6	72	3.7	73	3.8	77
Casualty	34	3.4	69	3.7	74	3.4	67	3.4	68	3.6	73
Dietician department	2	4.4	87	4.4	87	3.7	74	3.7	74	4.1	83
Maternity ward	16	3.7	74	3.2	65	3.7	74	4.0	79	4.2	84
Occupational health	2	4.1	81	4.5	90	3.8	76	4.1	81	3.9	77
Outpatient department	3	3.4	69	2.3	47	3.0	60	3.5	70	4.2	85
Paediatric ward	8	4.1	83	3.3	67	4.0	80	4.2	85	4.3	85
Pharmacy	12	3.5	71	3.6	72	3.3	67	3.4	68	3.9	78
Physiotherapy	2	3.9	77	2.2	44	4.0	80	4.5	90	4.6	93
Radiology department	6	4.1	83	4.0	80	3.6	72	4.0	79	3.9	77
Theatre	5	4.6	92	4.2	84	4.3	86	4.2	85	4.5	89
Thusong trauma centre	2	4.6	91	5.0	100	4.1	81	4.8	96	5.0	100
TOTAL	129	3.7	75	3.6	73	3.6	72	3.7	74	3.9	79

The biggest variance was seen in the leadership domain (Table 6), which ranged from 4.5 (90%) for occupational therapists to a low score of 2.2 (44%) for physiotherapists. Doctors consistently reported the lowest scores across most domains (team structure, situational monitoring, mutual support, and communication), verifying their overall score seen in Table 3.

The Thusong trauma centre recorded the highest scores, achieving two perfect means of 5.0 (100%) in the leadership and communication domains. The lowest scores recorded in the leadership domain were recorded by the outpatient (2.3; 47%) and physiotherapy (2.2; 44%) departments. Thus, leadership appears to be a vulnerable domain for these areas.

## Discussion

The overall mean perception of teamwork score among healthcare professionals at NDH was 3.7 (74%), indicating a generally acceptable level of teamwork (above 3; >60%).^20^ This finding is consistent with other reviewed studies.^19,20,21^ The only study found in the review to report an overall teamwork perception score of less than 60% focused on whether or not situating physicians and nursing staff in the same working station in an emergency department would improve communication and teamwork.^22^ However, the actual value of the results became clear by comparing the average scores for the teamwork domains, as well as analysing the differences between the different healthcare disciplines and departments.

### Domain strengths and weaknesses

Looking closer at the average scores for the different teamwork domains at NDH (Table 2), communication was identified as the strongest domain at NDH (3.9; 79%). This indicates staff are generally capable of interacting with one another and with patients in a timely and understandable manner. In comparison, NDH’s casualty communication score of 73% contrasts strongly with 52% in an emergency department reported in a similar study by Weaver et al.^22^, emphasising that results cannot be generalised.

Two areas for potential growth were identified: situational monitoring (72%) and leadership (73%). This implies staff members may have difficulty recognising important changes in their work environment, such as bed availability, changes in a patient’s condition, or a decrease in staff performance. This finding may also reflect a generalised lack of attention to detail and thoroughness within the clinical environment, which the 66 incomplete questionnaires may support. While the leadership domain speaks to a leader who is present, approachable, and leads by example, the 73% suggests a systemic opportunity to improve role clarity, task assignment, and psychological safety. These findings contrast with the diverse domain strengths and weaknesses found in other healthcare environments.^9,20,21,22^ For example, NDH’s Theatre team rated situational monitoring at 86%, far exceeding the 41% reported for an operating room in a study by Jonas et al.^21^

### Perceptions by discipline

Despite the variations in the mean scores among the healthcare disciplines at NDH (*n* = 128), no statistically significant difference was found (*p* = 0.434), possibly indicating that the observed differences are due to chance rather than systematic inter-disciplinary issues.

The lowest mean scores were recorded by doctors (70%) and pharmacy staff (71%). These are arguably the two disciplines with the most dynamic changes in the number and variety of patient contact. Appreciating this, both professions rated situational monitoring as their lowest teamwork domain (Table 5), stressing the importance of communicating changes in workload among team members. Conversely, the highest mean scores were reported by occupational therapists and dieticians. However, these results should be interpreted with caution due to the small sample sizes (*n* = 2). It was interesting to note that the two highest-scored domains for the two disciplines were leadership and team structure (Table 5), which may reflect the functional simplicity and clear roles typical of small, self-contained units.

A critical, localised finding emerged from the domain breakdown: physiotherapists (*n* = 2) reported the lowest score across the entire study in the domain leadership (2.2; 44%). This finding, despite their high communication score, suggests failure in perceived leadership within that specific unit, warranting investigation. This specific finding shows precisely why measuring teamwork perception is so valuable: it directs managers to critical areas needing immediate action.

### Perceptions by department

By comparing departments, while not achieving statistical significance (*p* = 0.066), a trend became clear that departmental context may influence teamwork perceptions.

Once again, the high-patient-turnover departments, the outpatient department and casualties, reported the lowest mean scores. Despite high communication scores (85%), the low leadership score (47%) in the outpatient department suggests staff feel they can communicate effectively but lack guidance or support from leadership. On the other hand, casualties displayed moderate leadership (74%) but appeared to lose sight of situational monitoring (67%), likely due to inherent patient pressure and flow in an emergency setting.

The Thusong trauma centre provided an example of positive deviance, recording the highest mean score (94%) and perfect scores in leadership and communication (100%, Table 6). This result may be attributed to the department’s small size, highly specialised purpose, and sense of mutual support necessary for dealing with sensitive patient issues.

### Limitations

Firstly, the high volume of excluded data must be acknowledged. Of the 195 returned questionnaires, 66 (33.8%) were excluded due to incompleteness (*n* = 62) or being returned empty (*n* = 4). The final analysis represents only 50.4% of the total target population (*N* = 256). The representativeness of the result may, therefore, have been affected by non-response bias. In the healthcare setting, individuals with high workloads or those who feel less engaged with their teams are often less likely to complete surveys. The findings might over-represent the views of those more motivated or satisfied, potentially skewing the teamwork perception scores. However, the diversity of the final sample suggests a broad range of professional perspectives.

Secondly, sample size and anonymity were concerns. Several disciplines and departments have only a few staff members. While measures were taken to protect confidentiality, this limited size may have introduced response bias and inhibited honest reporting, as participants may have been wary of identification. Thus, the outlier results observed in small groups (e.g. Thusong trauma centre, physiotherapy) must be interpreted with caution.

Thirdly, instrumental limitations also exist. The T-TPQ has not been formally validated in the South African healthcare context, potentially affecting reliability of these results.

Finally, the results are specific to the NDH context. The findings cannot be generalised to all hospitals in South Africa, as teamwork perceptions are known to vary across different levels of care (e.g. primary and secondary) and sectors (e.g. private facilities versus public facilities).

### Recommendations

From this study, the recommended focus for quality improvement must be directed towards the two weakest domains. To address the low leadership scores, training focusing on functions such as task assignment and effective delegation should be organised for managerial staff in the two lowest-scoring departments, specifically.^23^ A confidential management review should be conducted simultaneously to understand the structural factors contributing to these lower scores. To improve situational monitoring hospital-wide, NDH should introduce formal communication tools, such as TeamSTEPPS ‘Check-Backs’ and ‘Handoffs’, to verify information during high-risk transitions.^24^ This improvement in formal process links directly to the need for better data accuracy and thoroughness.

Additionally, the hospital should leverage its areas of excellence by using the Thusong trauma centre as an internal benchmark for positive teamwork. This could involve facilitating workshops led by Thusong staff to teach other departments the organisational factors that contribute to their effective teamwork.

Finally, we recommend that future research projects formally validate the T-TPQ tool for the South African context. Qualitative studies (focus groups or interviews) should also be conducted to explore the reasons for specific low scores reported by high-volume disciplines, and the extreme deficit observed in one specific discipline.

## Conclusion

This study successfully measured the perception of teamwork among healthcare professionals at NDH, finding an overall mean score of 3.7 (74%); thus, suggesting a generally acceptable level of teamwork. Despite the lack of a statistically significant difference in teamwork perception across disciplines (*p* = 0.434), the study’s main contribution is identifying specific areas where targeted improvement is needed. Hospital-wide, the potential areas for improvement were situational monitoring (72%) and leadership (73%). Unit-specific barriers were identified, particularly in the leadership domain in both the physiotherapy (44%) and the outpatient department (47%) units, suggesting a localised breakdown in perceived managerial support. On the other hand, the overall strongest domain was communication (79%). Also, the Thusong trauma centre (94%) served as an example of positive teamwork. Even while considering several limitations, such as the high rate of incomplete questionnaires (*n* = 66), the findings offer NDH management important, evidence-based data that can be used for targeted quality improvement.
